# Huge Mediastinal Thymic Cyst in the Elderly Patient

**DOI:** 10.7759/cureus.7240

**Published:** 2020-03-11

**Authors:** Behnam Shakerian, Mohammad Hossein Mandegar

**Affiliations:** 1 Cardiovascular Surgery, Shahrekord University of Medical Sciences, Shahrekord, IRN; 2 Cardiovascular Surgery, Tehran University of Medical Sciences, Tehran, IRN

**Keywords:** thymic cyst, mediastinum, huge, congenital

## Abstract

Mediastinal thymic cysts are uncommon lesions. Thymic cysts are usually diagnosed incidentally, and their origin could be congenital or acquired. Herein we present the case of a patient who presented with dyspnea. Chest computerized scan showed a large cystic mass. Surgical excision was performed. Pathology findings were consistent with congenital thymic cyst.

## Introduction

Thymic cysts are rare benign lesions. They constitute 1% of all mediastinal cysts [1]. They derive from a patent thymopharyngeal duct. Because of their infrequent occurrence, preoperative diagnosis is rarely achieved. Preoperative diagnosis depends on an imaging examination. However, it is difficult to distinguish thymic cysts from solid neoplasms of the thymus by imaging examination in some cases [2]. This lesion is uncommon in the elderly, and there is a male predominance of almost 3:1 [3]. Most patients are asymptomatic. The treatment of choice is complete resection of the cyst. The purpose of surgery is to make a definitive diagnosis and to prevent or treat complications, such as rupture, compression of the surrounding organs, and malignant transformation.

## Case presentation

**Figure 1:**
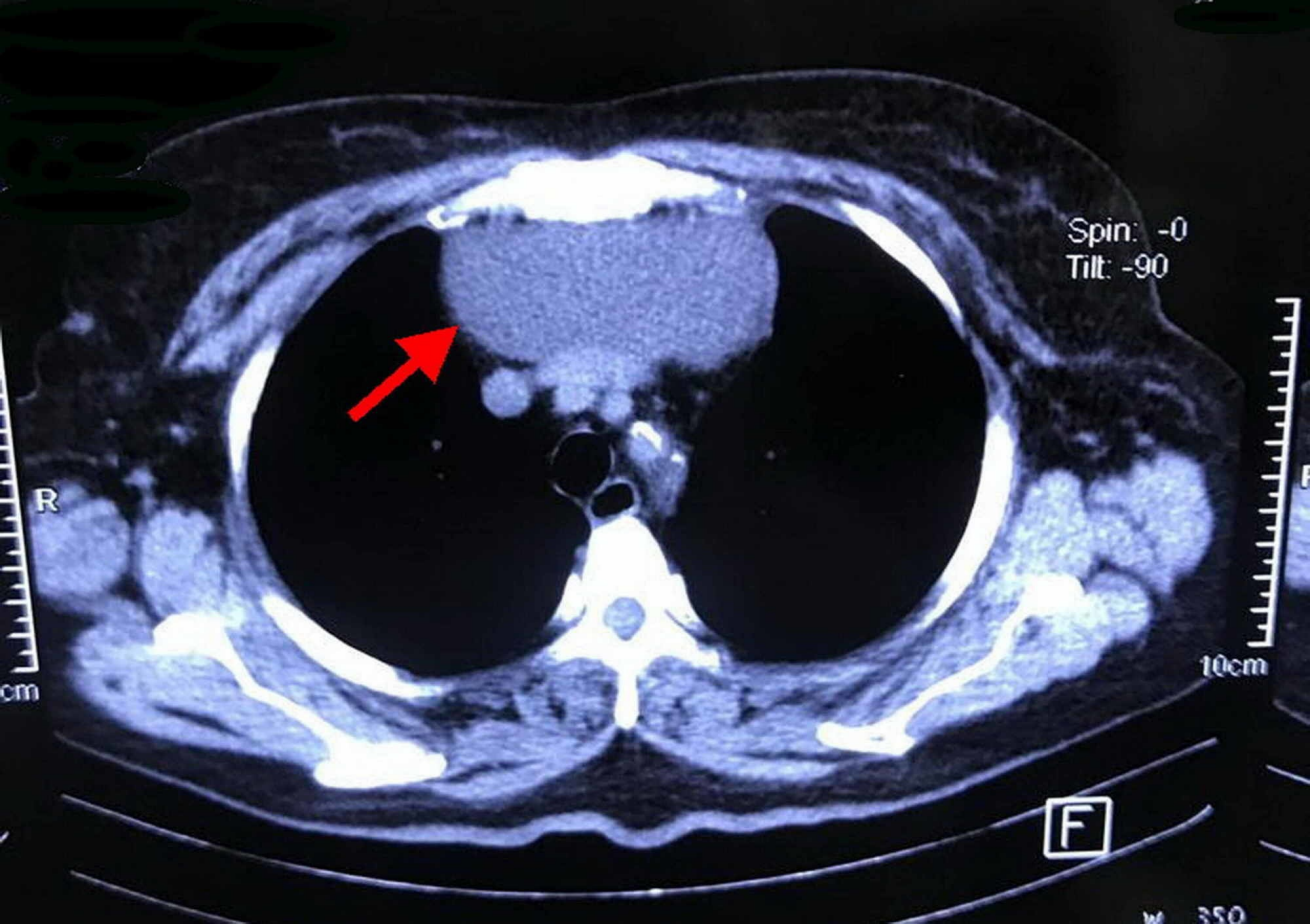
CT of the chest showing a cystic mass in mediastinum (red arrow).

A 67-year-old woman with a history of closed mitral valve commissurotomy carried out via right thoracotomy 20 years ago present with a 10-month history of dyspnea. Cardiac examination demonstrated an irregularly irregular pulse with a mid-diastolic rumbling murmur at the apex. She demonstrated one plus pitting edema of the lower extremities. Echocardiography showed a calcified rheumatic mitral valve, severe mitral stenosis, moderate tricuspid regurgitation, and loculation in the lung filed. A chest X-ray revealed a radio dense mass in the mediastinum. Computed tomography (CT) of the chest showed a cystic mass in the mediastinum that was about 10.70 × 5.35 cm in size and extended from the upper end of the sternum to 11.52 cm below it (Figure 1).

**Figure 2:**
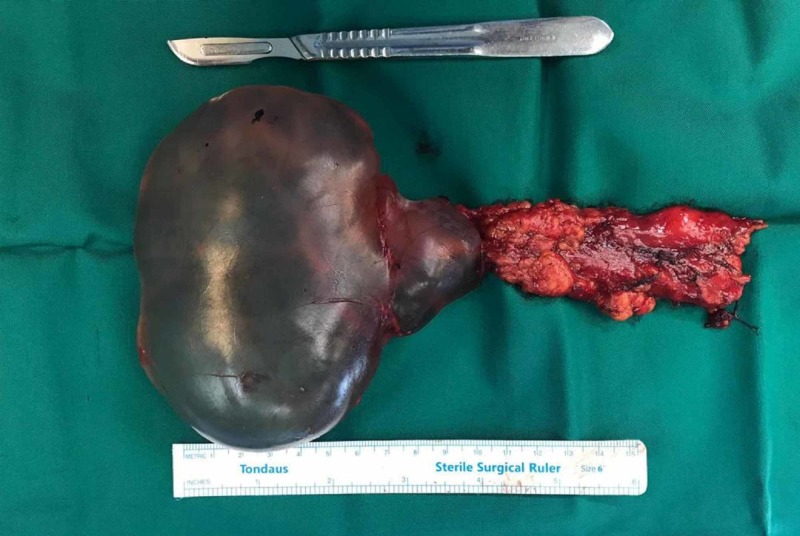
Surgical view of the thymic cyst.

A coronary angiography did not reveal coronary obstruction. She was scheduled for mitral valve replacement, tricuspid valve repair, and excision of the mediastinal cyst. During sternotomy, a cystic lesion was found in the middle mediastinum. The cyst was well defined and encapsulated and had a thin wall without any signs of invasion to the surrounding tissue. The cystic lesion was excised with thymic remnant without rupture and sent for pathological examination. The surgical specimen was a unilocular cyst with a smooth surface and clear content measuring 10×10×5 cm and weighing 236 g (Figure 2).

**Figure 3:**
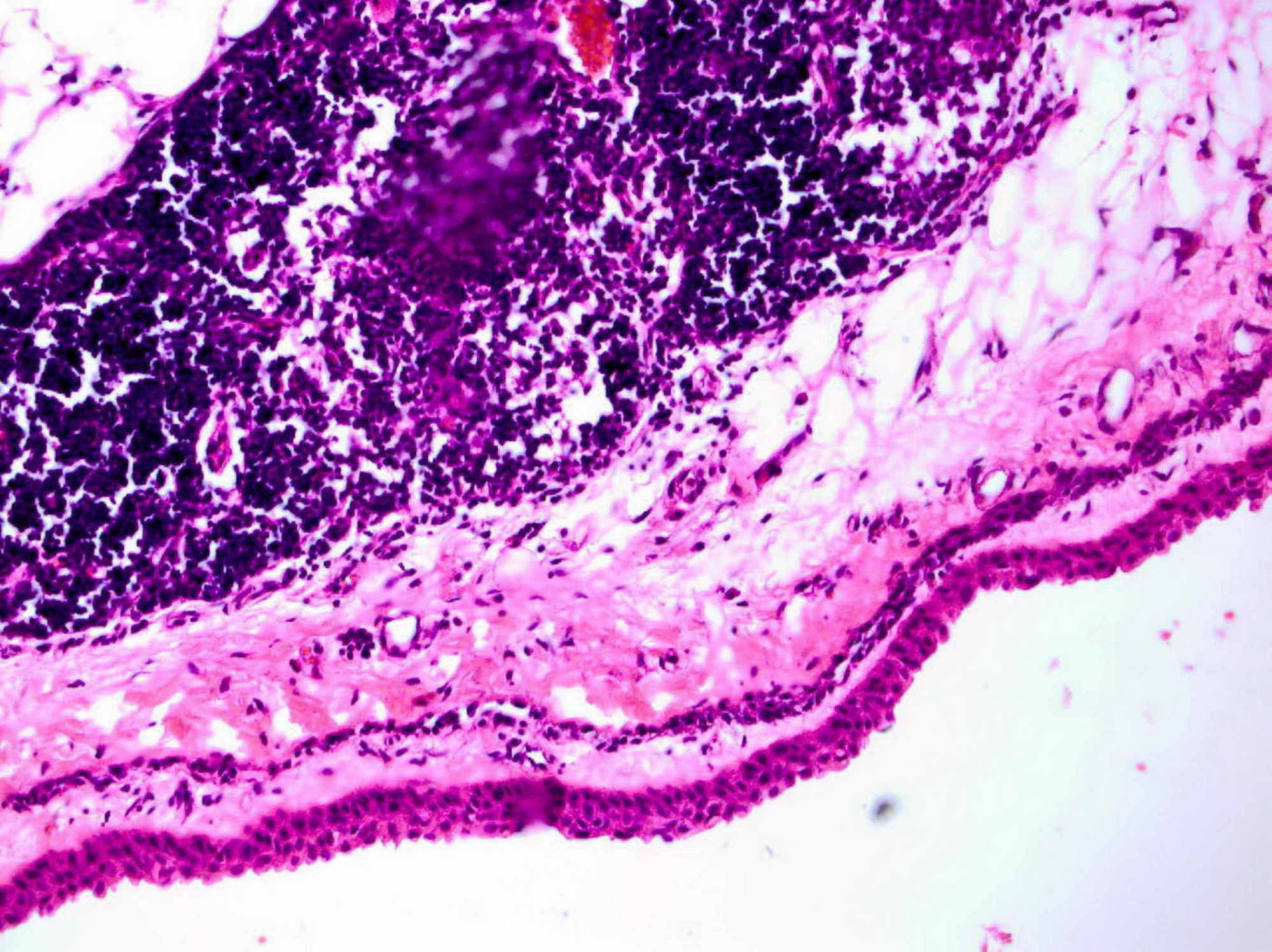
Histological image of the thymic cyst.

Microscopic examination showed a cystic lesion filled with a clear material with a maximum wall thickness of 0.2 cm compatible with a unilocular thymic cyst (Figure 3).

 The patient was discharged without any problems on the seventh day after the operation.

## Discussion

A mediastinal thymic cyst is a very rare pathological finding comprising only 1% of all mediastinal cysts. Thymic cysts were first defined by Lieutaud in 1832 [4]. Thymic cysts are most commonly found in the pediatric patients, but they can be seen in all age groups [5]. However, they can also occur very rarely at advanced ages. Most of them are located in the anterior mediastinum. However, they may be localized anywhere along the normal descent route of the thymus gland from the base of the neck to the diaphragm. They can be either acquired or congenital. Congenital cysts are usually unilocular with a thin wall and clear liquid content, representing derivatives of the embryonal thymic tissue. The acquired type is usually multiloculated with a thick and fibrous wall. Acquired thymic cysts are associated with infection, autoimmune diseases, neoplasia, radiation therapy, and thoracotomy. Thymic cysts are usually asymptomatic and incidentally detected on a chest X-ray, CT, or during surgery [6]. Symptoms appear late when the cyst produces pressure effects. The common symptoms include dyspnea, chest pain, cough, hoarseness, and dysphagia. The cysts can enlarge rapidly as a result of hemorrhage within the cyst or infection. A hemorrhagic thymic cyst can also cause hemothorax. A CT scan with contrast may aid in the diagnosis of thymic cysts. A fine needle aspiration biopsy is very valuable for cytology, but has not proven useful in the differential diagnosis of thymic cysts [7]. The differential diagnosis of thymic cysts includes thymoma, lymphangioma, lymphoma, hemangioma, and pericardial or bronchogenic cysts. The current treatment of thymic cysts is still controversial. Some believe that all thymic cysts should be surgically removed for a definitive diagnosis and cure. Some others believe that treatment depends on the symptomatology, cyst size, and associated pathologies.

## Conclusions

Mediastinal thymic cysts in the elderly patients are extremely rare. Surgical resection results in definitive diagnosis. Prognosis for thymic cyst is good.
